# American martens use vigilance and short-term avoidance to navigate a landscape of fear from fishers at artificial scavenging sites

**DOI:** 10.1038/s41598-021-91587-4

**Published:** 2021-06-09

**Authors:** Todd M. Kautz, Dean E. Beyer, Zachary Farley, Nicholas L. Fowler, Kenneth F. Kellner, Ashley L. Lutto, Tyler R. Petroelje, Jerrold L. Belant

**Affiliations:** 1 Global Wildlife Conservation Center, State University of New York, College of Environmental Science and Forestry, 1 Forestry Drive, Syracuse, NY 13210 USA; 2 Wildlife Division, Michigan Department of Natural Resources, 1990 US Highway 41 S, Marquette, MI 49855 USA; 3 Department of Fish, Wildlife, and Conservation Ecology, New Mexico State University, College of Agricultural, Consumer, and Environmental Sciences, 2980 South Espina, Las Cruces, NM 88003 USA

**Keywords:** Behavioural ecology, Boreal ecology, Community ecology, Animal behaviour

## Abstract

Where two sympatric species compete for the same resource and one species is dominant, there is potential for the subordinate species to be affected through interference competition or energetic costs of avoiding predation. Fishers (*Pekania pennanti*) and American martens (*Martes americana*) often have high niche overlap, but fishers are considered dominant and potentially limiting to martens. We observed presence and vigilance of fishers and martens at winter carcass sites using remote cameras in Michigan, USA, to test the hypothesis that interference competition from fishers creates a landscape of fear for martens. Within winters, fishers co-occupied 78–88% of sites occupied by martens, and martens co-occupied 79–88% of sites occupied by fishers. Fishers displaced martens from carcasses during 21 of 6117 marten visits, while martens displaced fishers during 0 of 1359 fisher visits. Martens did not alter diel activity in response to fisher use of sites. Martens allocated 37% of time to vigilance compared to 23% for fishers, and martens increased vigilance up to 8% at sites previously visited by fishers. Fishers increased vigilance by up to 8% at sites previously visited by martens. Our results indicate that fishers were dominant over martens, and martens had greater baseline perception of risk than fishers. However, fishers appeared to be also affected as the dominant competitor by putting effort into scanning for martens. Both species appeared widespread and common in our study area, but there was no evidence that fishers spatially or temporally excluded martens from scavenging at carcasses other than occasional short-term displacement when a fisher was present. Instead, martens appeared to mitigate risk from fishers by using vigilance and short-term avoidance. Multiple short-term anti-predator behaviors within a landscape of fear may facilitate coexistence among carnivore species.

## Introduction

Interference competition occurs when a dominant species prevents a subordinate species from accessing a shared resource by direct physical confrontation^1^. Interference competition is common among carnivores at concentrated foraging sites such as carcasses^2,3^, with subordinate species having greater risk of injury or mortality than dominant species during competitive encounters (e.g., intraguild predation^4^). Consequently, subordinate carnivore species face a trade-off where encounters with dominant species are dangerous but avoiding dominant species may result in loss of access to high-quality resources^5^. along with other energetic costs such as vigilance and escape^6^.

The landscape of fear hypothesis proposes that animals perceive a spatial “topography” of predation risk in their environment^7^, which may vary temporally^8,9^. Animals can respond to perceived predation risk by avoiding feeding in risky areas^10^, using high-risk areas when predators are inactive^9,11^, or increasing anti-predator behaviors (e.g., vigilance^12^). However, currently there is limited research that identifies precise spatial and temporal scales at which prey perceive and respond to predation risk^13,14^. Understanding the scales at which subordinate carnivores perceive and respond to risk from dominant competitors can further explain the complex competitive interactions among carnivore species.

Competition at carcasses may be important for mustelids, which often derive a large proportion of their diet from carrion^3^. American martens (*Martes americana*; hereafter martens) and fishers (*Pekania pennanti*) are mesocarnivores that compete when sympatric due to high niche overlap^15,16^. Adult fishers typically weigh 2.0–5.5 kg with home range size that varies with habitat but is usually 15–35 km^2^^17^. Martens are smaller with typical body mass of 0.4–1.0 kg and a home range of 2–8 km^2^^17^. Though fishers can kill larger prey, fishers and martens generally have high dietary overlap and carrion is an important food source for both species^17^. When encounters occur, the larger fisher is dominant and can kill martens^18–20^. In some cases, competition and intraguild predation from fishers may regulate marten populations^15,16^. Martens may temporally alter their use of localized resources after fisher visitation^21^, suggesting that martens may detect fisher scent or other cues and adjust their avoidance behavior. Aspects of marten-fisher relationships that have not been examined include the temporal scales at which martens perceive risk from fisher presence or fisher cues, and the role of fine-scale behavioral risk responses such as short-term avoidance and vigilance.

We investigated vigilance behavior and interference competition between fishers and martens at artificial scavenging sites during winter. We hypothesized that interference competition from dominant fishers creates a landscape of fear for martens, and martens exhibit behavioral risk responses that reduce loss of access to carcasses. We predicted that: (1) fishers, as the more dominant carnivore, would allocate less time toward vigilance than martens at carcasses, (2) fishers would displace martens from carcasses while martens are feeding, but martens would not displace fishers from carcasses while fishers are feeding, (3) fisher visitation to carcasses would cause marten risk responses, with martens favoring fine-scale responses (increased vigilance and short-term avoidance) over responses with greater loss of access to carcasses (altered diel activity and long-term avoidance), and (4) marten visitation to carcasses would cause fishers to increase vigilance as fishers exploit opportunities to kill or injure martens.

## Results

### Scavenging detections

We recorded 10,277 fisher images (1743, 3495, and 5039 during 2017, 2018, and 2019, respectively) and 28,770 marten images (6057, 13,080, and 10,404 during 2017, 2018, and 2019, respectively). We detected 6117 independent marten visits, and 1359 independent fisher visits. We detected martens in pairs in 743 images during 232 independent visits (5% of marten images, 3.8% of marten visits), and in 1 visit we observed 3 martens together. All observed fishers were solitary.

**Figure 1:**
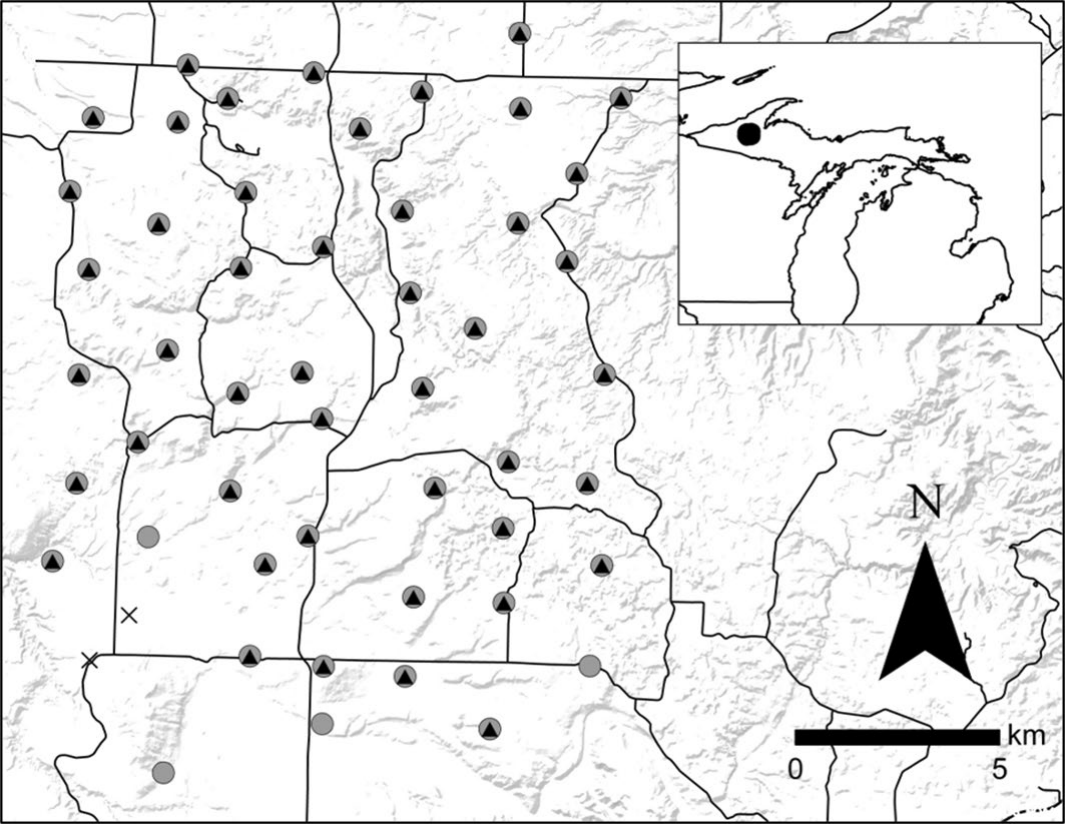
Carcass sites (*n* = *52*) used to monitor American marten and fisher activity and behavior in Upper Peninsula of Michigan, USA, January–March 2017–2019. Triangles represent sites with martens detected, circles represent sites with fishers detected, Xs represent sites with neither species detected, and black lines represent roads. Map generated using QGIS (QGIS Development Team 2020, version 2.14.20; available from http://qgis.osgeo.org).

For the combined 3 winters, fishers co-occupied 100% of sites occupied by martens (47 of 47 sites), while martens co-occupied 94% of sites occupied by fishers (47 of 50 sites; Fig. 1). Within winters, fishers co-occupied 78–88% of sites occupied by martens, while martens co-occupied 79–88% of sites occupied by fishers.

Although sites were designed to attract bobcats, we observed bobcats only within 7 of 156 seasonal sessions (52 sites with 3 winters per site). We observed no wolves, coyotes, red foxes (*Vulpes vulpes*), or gray foxes (*Urocyon cinereoargenteus*), although these species occurred in the study area. We observed large avian predators at carcasses, including bald eagles (*Haliaeetus leucocephalus*), golden eagles (*Aquila chrysaetos*), barred owls (*Strix varia*), ravens (*Corvus corax*), and American crows (*C. brachyrhynchos*). Due to mechanical failures or snow covering lenses, cameras were inoperable for 378 of the 8,736 site-days surveyed (4.3% inoperable rate). The maximum continuous period a camera was inoperable was 14 days.

### Interference displacement events

**Figure 2:**
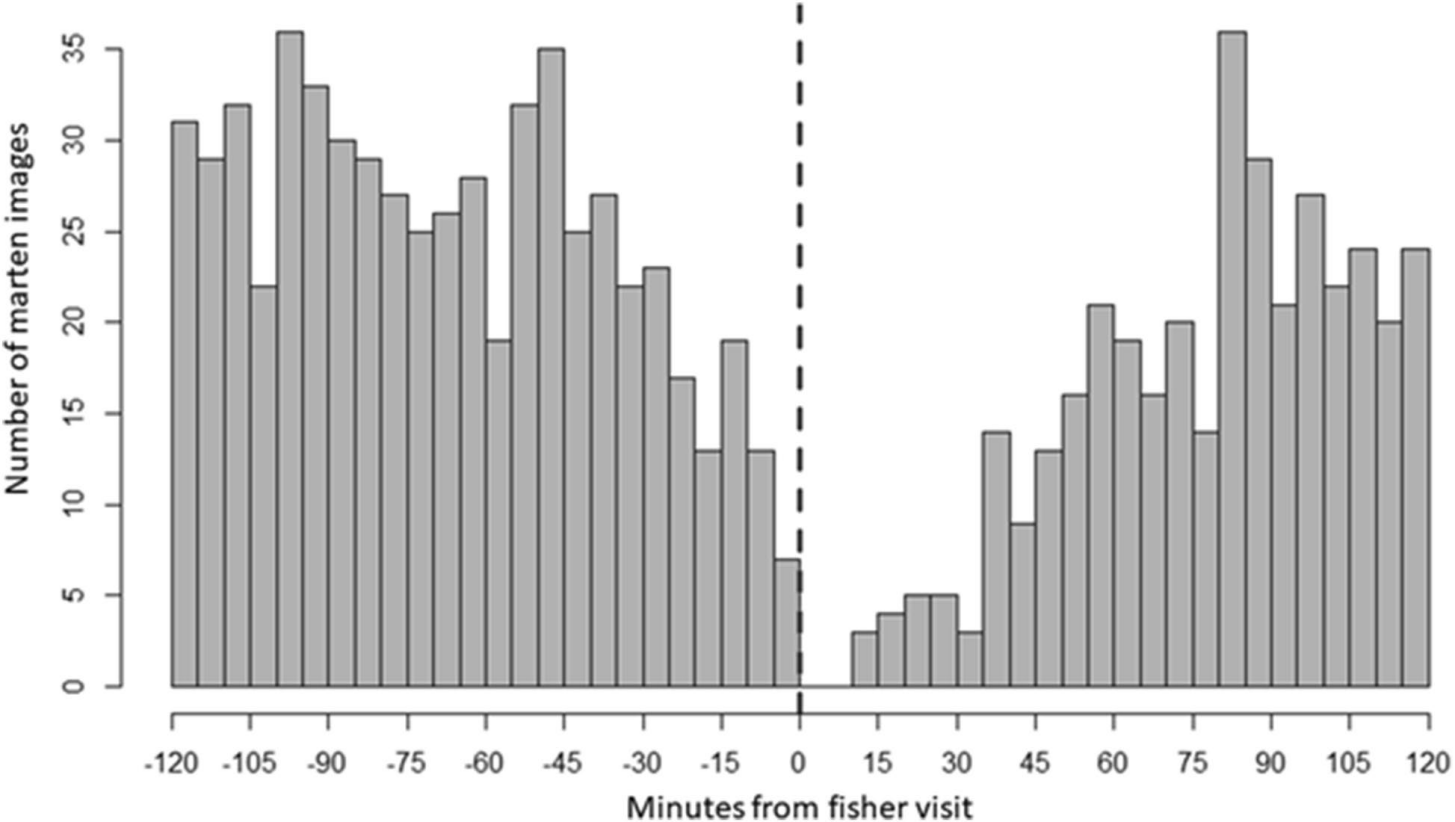
Histogram of American marten images recorded by remote cameras at scavenging sites within 2 h before (left of vertical dashed line) and after (right of vertical dashed line) fisher presence (vertical dashed line), Upper Peninsula of Michigan, USA, January–March 2017–2019.

During 6117 independent marten visits, we observed 21 instances where fishers apparently displaced martens. During 1359 independent fisher visits, we observed 0 instances of martens displacing fishers. The apparent rate of fishers displacing martens was 1 per 291 marten visits (95% CI 1:192–1:476), while the apparent rate of fishers displacing martens was 0 (95% CI 0–1:370; *P*[marten displacement > fisher displacement] = 0.023). The distribution of marten images within 2 h of fisher use of sites suggests martens used sites at a reduced frequency within 15–20 min of fisher presence and within 80 min martens used sites at a similar frequency to periods before fisher arrival (Fig. 2). Additionally, marten use of sites began to decline about 30 min before fisher arrival, suggesting some martens detected fishers and left sites > 10 min before fisher arrival.

### Vigilance responses

We obtained 9195 fisher images and 25,463 marten images where behavior could be classified. Fishers were vigilant in 23% of images (95% CI 22–24%), with the remaining images 70% feeding and 7% other non-vigilant). Martens were vigilant in 37% of observations (95% CI 36–37%) with the remaining images 57% feeding and 6% other non-vigilant. Martens observed in pairs were vigilant in 29% of observations (*n* = 1168, 95% CI 26–32%), while solitary martens were vigilant in 37% of observations (*n* = 24,267, 95% CI 36–38%).

**Figure 3:**
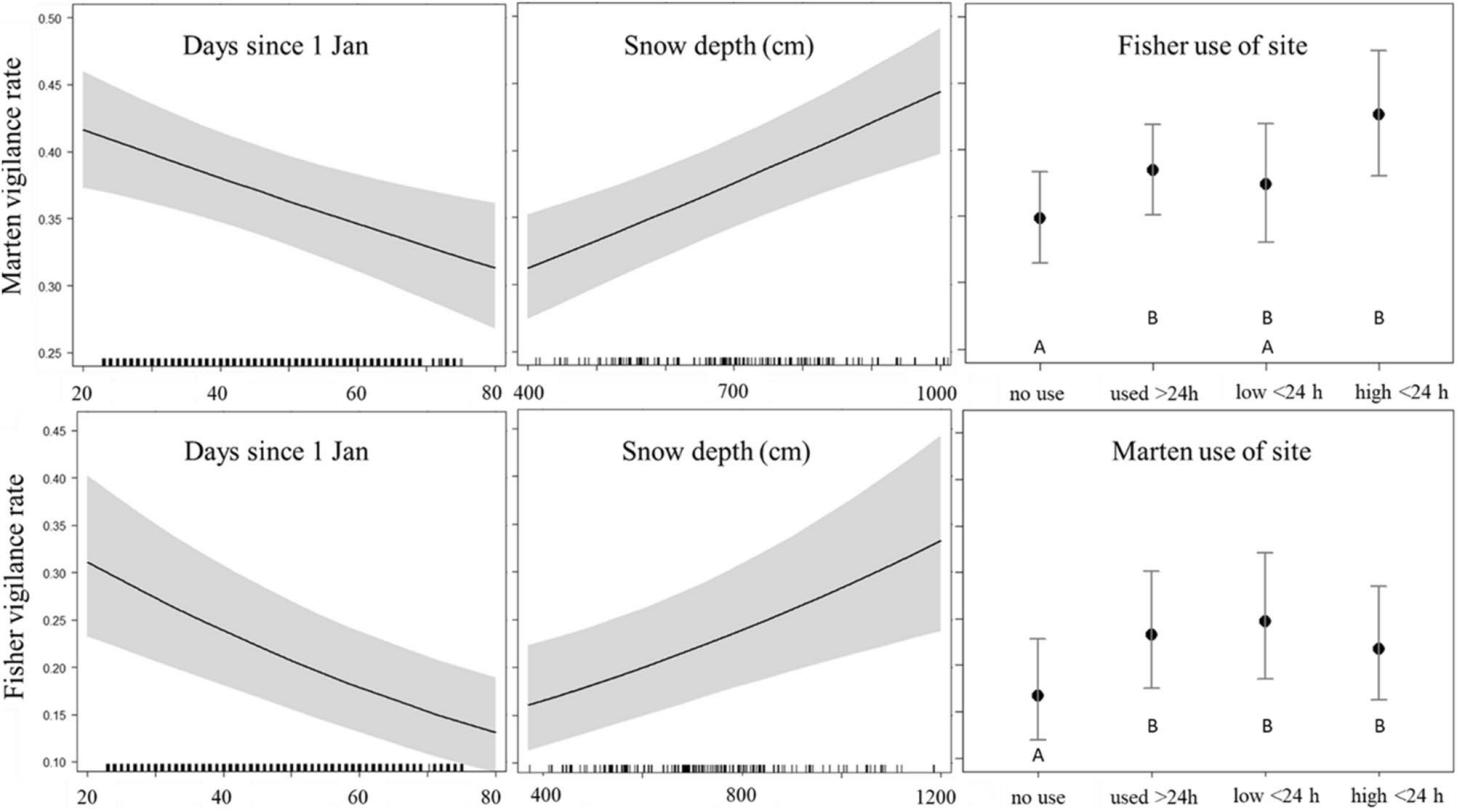
Logistic regression effect estimates with 95% confidence intervals for factors influencing probability of vigilance behavior for American marten (top row) and fisher (bottom row), Upper Peninsula of Michigan, USA, January–March 2017–2019. Interspecific site use classifications (right column) include: “no use” as sites with no previous observations of the other species within the winter, “used > 24 h” as sites with the other species previously observed but not within the previous 24 h, “Low < 24 h” as sites with median or less image counts of the other species within the previous 24 h, and “high < 24 h” as sites with greater than median image counts of the other species within the previous 24 h. Letters represent statistically different estimates (*P* < 0.05).

We used and 7086 fisher images and 17,870 marten images collected after 22 January for logistic regression estimating how fisher and marten vigilance responded to previous site use by the other species (Table 1). Fishers increased vigilance from 17% at sites without previous marten use observed to 22–25% (P < 0.05) at sites with previous marten use and were similarly vigilant whether marten use occurred within the past 24 h or was of low or high intensity (Fig. 3). Martens spent 35% of time vigilant at sites not previously used by fishers, but increased vigilance to 43% with high fisher use within the past 24 h (*P* < 0.001) and 38% with previous fisher use > 24 h prior (*P* < 0.001), but lacked strong support for increased vigilance at sites with low fisher use within the past 24 h (38%; *P* = 0.139). In all cases, fishers and martens trended towards increased vigilance at sites with previous use by the other species (Fig. 3). Fishers and martens increased vigilance in response to increasing snow depth and decreased vigilance in response to increasing days since 1 January (Fig. 3, Table 1).

**Table 1:** Logistic regression fixed effect estimates with 95% for factors influencing probability of vigilance behavior for American martens and fishers, Upper Peninsula of Michigan, USA, January–March 2017–2019. For each species, occasions with no previous observations of the other species were estimated within the intercept. Both models included year of survey as a random effect.

Predictor	β	se	*Z*	*P*	*n* images
**Marten vigilance**					
Intercept (no previous fisher use)	− 0.970	0.115	− 8.431	< 0.001	4804
Days since 1 January	− 0.007	0.002	− 3.195	0.001	NA
Snow depth (cm)	0.094	0.020	4.602	< 0.001	NA
High fisher use < 24 h	0.331	0.076	4.361	< 0.001	884
Low fisher use < 24 h	0.111	0.075	1.478	0.140	1003
Fisher use > 24 h	0.157	0.038	4.102	< 0.001	11,179
**Fisher vigilance**					
Intercept (no previous marten use)	− 1.617	0.237	− 6.807	< 0.001	814
Days since 1 January	− 0.018	0.008	− 4.584	< 0.001	NA
Snow depth (cm)	0.115	0.032	3.567	< 0.001	NA
High marten use < 24 h	0.489	0.114	4.283	< 0.001	1.411
Low marten use < 24 h	0.323	0.112	2.880	0.004	1810
Marten use > 24 h	0.410	0.104	3.930	< 0.001	3051

### Circadian activity responses

**Figure 4:**
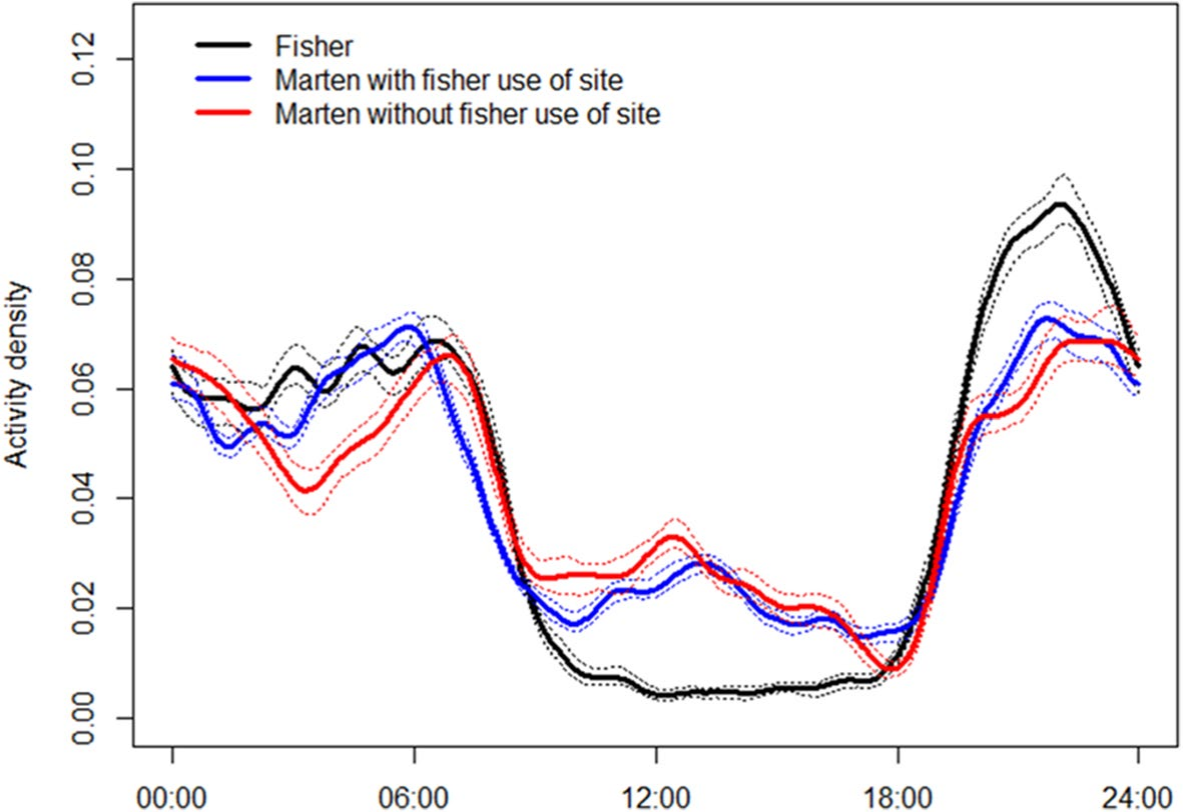
Circular kernel density estimates of diel activity with 95% confidence intervals (dotted lines) for fisher and American marten at sites with previous use by fisher or no previous use by fisher within the same winter, Upper Peninsula of Michigan, USA, January–March 2017–2019.

After 22 January we obtained 7919 fisher images, 15,464 images of marten at sites with previous fisher observations within the winter, and 5420 images of martens at sites without previous fisher observations within the winter. Marten activity was nearly identical with and without previous fisher observation (overlap = 0.92, 95% CI 0.90–0.95), and activity overlap between martens and fishers at sites with fisher observation (overlap = 0.87, 95% CI 0.84–0.90) was similar to marten and fisher activity overlap at sites without fisher observation (overlap = 0.84, 95% CI 0.81–0.87; Fig. 4).

## Discussion

Our hypothesis that interference competition from dominant fishers created a landscape of fear for martens was generally supported; however, our results also suggest that marten risk responses to fishers occurred at fine temporal scales and constraints on marten foraging were limited. Our prediction that fishers would allocate less time towards vigilance than martens while using carcass sites was well supported; martens devoted 14% more time (proportionally, a 1.61-fold increase) to vigilance compared to fishers. Additionally, we found support for our prediction that fishers would displace martens from carcasses while martens never displaced fishers. Together, these results support that fishers were dominant and martens had greater baseline perception of predation risk.

Supporting our predictions, martens were more vigilant (up to 8%) with fisher visitation at carcasses relative to sites not previously used by fishers. As we are unaware of previous records of American marten vigilance for comparison, the ecological importance of this effect is uncertain. However, this suggests potential energetic costs for martens with increasing risk of fisher encounter. Though statistical support was marginal, our results suggest that recent and intense fisher site use had a larger effect on marten vigilance. Other studies have generally found no vigilance response of free-ranging mesocarnivores to the presence of dominant carnivores^22,23^, although experimental studies found increased vigilance of mesocarnivores in response to presence and scent cues of dominant carnivores^24^. Our results provide evidence that mesocarnivore vigilance can be influenced by dominant carnivore presence in a wild population.

A short-term avoidance response of martens to fishers was also supported but was limited to about 80 min after fishers left carcass sites. Because fishers only displaced martens in 1 per 291 marten visits, this suggests that martens had minimal loss of foraging opportunity due to short-term avoidance of fishers. Similar to a nearby study by Croose et al.^25^, we found no evidence that martens altered their diel activity to avoid fishers; however, there was some temporal niche partitioning apparent where martens were more active than fishers during daylight. Finally, spatial overlap among fishers and martens was high, demonstrated by high (> 75%) co-occupancy of the same carcass sites and most observations of both species occurring at carcass sites previously visited by the other species. Therefore, although both species appeared widespread and common in our study area, there was little evidence that fishers spatially or temporally excluded martens from scavenging at carcasses at a coarse scale. Martens have high metabolic rate and low energy storage which requires daily foraging^26^, so the nutritional benefits afforded martens by carcasses may have overridden predation risk from fishers unless a fisher was present.

Fishers were more vigilant at sites with previous marten use, though it did not appear important whether marten use was recent or more intense. It is unlikely that fisher vigilance in response to martens was a result of fear, as martens never displaced fishers from carcasses and fishers are predators of martens^18–20^. Vigilance is often interpreted as a predation risk response, but our results suggest that vigilance may also reflect alertness in a dominant competitor based on the chances of encountering a subordinate competitor. There are several possible reasons for a dominant predator to become more vigilant in response to scent from a subordinate species. Mammalian predators can be attracted and intensify search behavior in response to olfactory cues of nearby prey^27,28^, and fishers may have had an aggressive response seeking opportunities to predate martens. Alternatively, fishers may have had a defensive response and were scanning to prevent martens from coming nearby and potentially consuming parts of the carcass. Though our carcass sites were artificially contained to within a few square meters, naturally occurring large carcasses often are disarticulated and dispersed over larger areas during scavenging^29^, in which case fishers may despotize more resources by scanning for nearby martens attempting to use disjunct portions. Our results suggest martens were not deterred from carcasses by fishers except when fishers were present, so fishers may have sought opportunities for direct confrontation or predation if those were the only effective ways to decrease sharing of resources with martens. Finally, marten scent may have elicited an exploratory or curiosity response from fishers, similar to mesocarnivores such as red fox which respond to conspecific scent by spending more time investigating their surroundings (Leo et al. 2015). Regardless of the reason for fisher vigilance response, our results support the hypothesis that maintaining an exclusive resource patch requires effort from the dominant competitor which may have energetic costs^30^.

Outcomes of competition between martens and fishers are variable. For example, martens and fishers may co-occur but spatially segregate in winter where martens favor areas with deeper snow^18,31,32^. In contrast, sympatric martens and fishers may avoid each other by using different habitats^15,33^ or by martens temporally avoiding fishers^21^. Where martens and fishers exhibit high spatio-temporal overlap, martens may be limited by fisher competition^16^ or coexist with fishers with little apparent response as we observed in this study^25^. Our results suggest marten vigilance as a possible mechanism for coexistence with fishers despite high niche overlap. Deep snow conditions within our study may have facilitated the ability of martens to flee from approaching fishers, because martens have > 2 times lighter foot loading than fishers and are better adapted to moving over deep snow^34^. However, actual vs perceived predation risk are often conflated in landscapes of fear studies^13^, and we emphasize that we measured fear-based behavioral responses of martens, not actual predation risk. Despite this limitation, there was an apparent abundance of martens and fishers at our study sites and a history of sustainable fur harvest for both species in the region^35^, suggesting that martens have been successful in our study area despite the presence of fishers.

We detected several trends in marten and fisher vigilance unrelated to our hypothesis. The reduction in vigilance for martens feeding in pairs is consistent with the hypothesis that group vigilance can partially compensate for individual vigilance^6,23,36^. However, because > 95% of marten visits were solitary individuals, foraging in groups is not likely an important risk response for martens. Additionally, marten and fisher vigilance had similar positive relationships with snow depth and negative relationships with day of year. Deeper snow could increase vigilance by accumulating above the carcass and filling in brush walls, limiting marten and fisher peripheral vision while feeding and necessitating them to raise their heads to scan more often. If this holds, the effect of snow depth could represent an artifact of our site design and not an ecological process. Decreasing vigilance with day of year could have been a consequence of site familiarity if animals made repeated visits, which may reduce predation risk^37^. Our interpretation is limited as we included snow depth and day of year as control variables, but vigilance effect estimates for these variables were considerable (e.g., > 50% reduction in fisher vigilance from beginning to end of survey; Fig. 3) and may warrant further study.

A limitation of our study was that sites were baited 2–2.5 weeks before cameras were deployed. Consequently, sites that we attributed as “not used” by fishers and martens could have been visited before cameras were deployed. Because we excluded observations before 22 January, errors in the “not used” site classes due to missed early monitoring were unused for at least 14 days; however, it is possible that heterospecific scent cues influenced fisher and marten behavior beyond 14 days. Inoperable camera sites could also have contributed error, but this was probably minimal with < 5% inoperable rate. The respective vigilance responses of martens and fishers to previous site use by the other species may have been stronger than our results suggest if some observations in the “not used” class were influenced by > 14 day-old scent cues from unobserved animals. The effects of missed observations on co-occupancy estimates were probably small because observed co-occupancy was high and most sites visited by fishers or martens before cameras were deployed likely had subsequent fisher detections recorded on camera. For example, of sites with fishers detected within the first 2 weeks of the 8-week period when cameras were deployed each winter, 59% had fisher detections during week 3 and 86% had fisher detections by week 8. Consequently, we suggest we adequately characterized fisher-marten interactions, but our inference was limited to 8 weeks after carcasses had been available, but not monitored for 2–2.5 weeks.

By excluding canids, the artificial scavenging sites in our study did not represent the scavenging guild at natural carcasses. Within our study area, wolves, coyotes, red foxes, gray foxes, and bobcats are potential predators or dominant competitors of fishers and martens^32,38–41^, but were largely or entirely absent from our carcass sites. Abundant bobcat detections in nearby surveys using the same hair snare methods suggests that the scarcity of bobcats reflected low bobcat density^42,43^. However, concurrent population estimates within our study area suggested that coyotes and wolves were common and deliberately avoided these sites (Jerrold Belant, unpublished data). Consequently, our study did not represent competitive interactions at naturally occurring scavenging sites but offered the advantage of examining fisher and marten interactions with minimal confounding effects of carcass visitation by other dominant mammalian carnivores. Finally, remote cameras can elicit reactions from animals that include wariness and aversion^44^. Fishers and martens in our study were exposed to the same cameras but we did not know whether they had similar reactions to cameras, which could have influenced respective vigilance rates.

Evidence of subordinate species using fine-scale behavioral risk responses instead of broad-scale avoidance has implications for carnivore competition. For example, although gray wolves are dominant over and sometimes kill coyotes during interference competition interactions^45,46^, coyotes and gray wolves in the Great Lakes region coexist with considerable spatial, temporal, and dietary niche overlap^47,48^. Coyotes respond to wolf risk through increased vigilance when using shared resources such as carcasses^49^, so coyote vigilance could in part explain coyote-wolf coexistence. More broadly, dominant carnivores often, but not always, tend to limit subordinate carnivore abundance^3^. Where demographic or landscape-scale carnivore competitive responses are unexpectedly absent, short-term behavioral responses may be a mechanism facilitating coexistence^50^. Though many studies of vigilance have focused on herbivorous prey^51–55^, vigilance may also be an important risk response to intraguild predators and competing carnivore species^23,56,57^.

## Methods

### Study area

Our study was conducted during January–March 2017–2019 in a 260-km^2^ area of the Upper Peninsula of Michigan, USA (46.6° N, 88.89° W) (Fig. 1). Land cover comprised deciduous forests (29%), woody wetlands (11%), mixed forests (31%), conifer forests (22%), open water (< 1%), grassland/herbaceous (< 1%), developed (< 1%), and other (< 5%; 2016 National Land Cover Database^58^). Dominant tree species include sugar maple (*Acer saccharum*), eastern white pine (*Pinus strobus*), trembling aspen (*Populus tremuloides*), eastern hemlock (*Tsuga Canadensis*), black spruce (*Picea mariana*), and northern white cedar (*Thuja occidentalis*). The study area has no major highways and is interspersed with secondary roads, most of which are passable only with snowmobiles during winter. Snowfall ranges from 225 to 400 cm (National Oceanic and Atmospheric Administration 1981–2010; https://www.ncdc.noaa.gov/cdo-web/datatools/normals summary). Average daily minimum temperature during the study was -14.7 °C (PRISM Climate Group temperature estimates; https://prism.oregonstate.edu).

### Artificial scavenging sites

**Figure 5:**
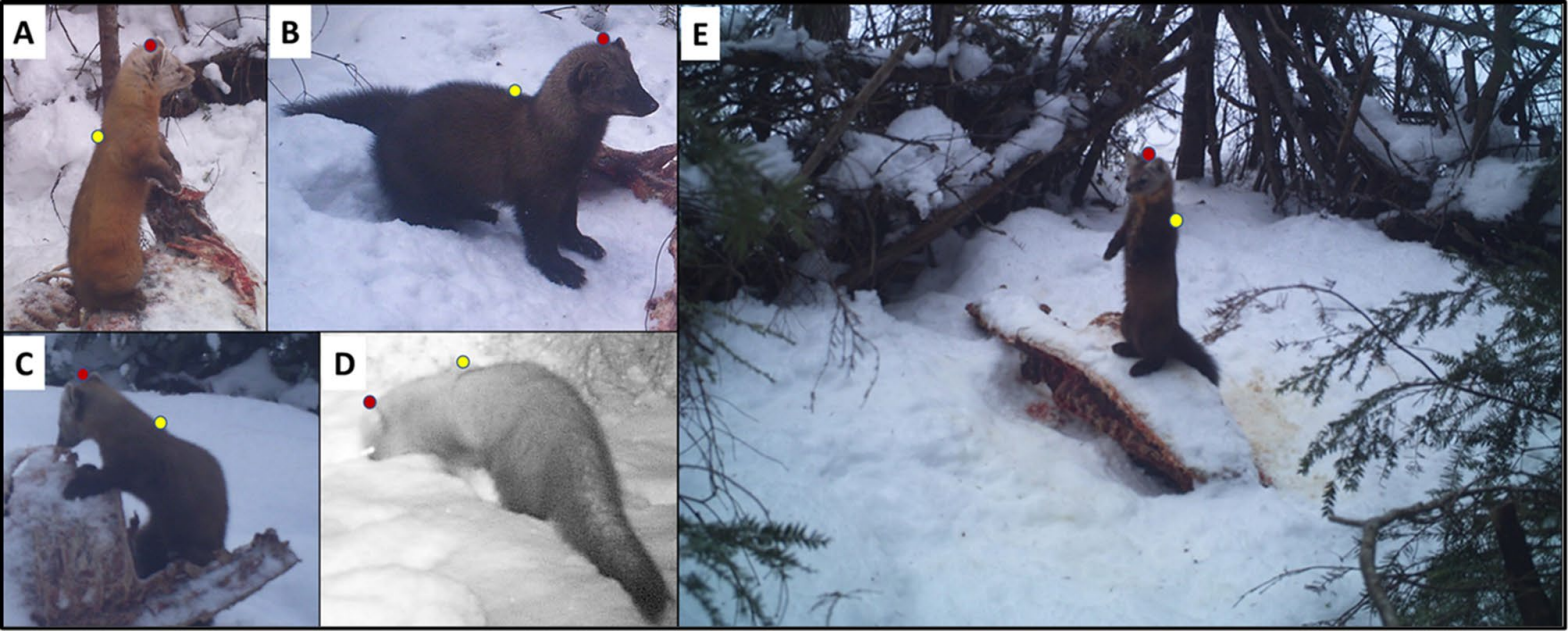
Behavioral classifications of American martens and fishers as examples of (**A**) vigilant marten, (**B**) vigilant fisher, (**C**) feeding marten, (**D**) non-vigilant fisher, and (**E**) vigilant marten at site with partial white-tailed deer carcass and brush enclosure visible. Red and yellow dots represent the top of the head and top of the shoulders, respectively, used to assess vigilance posture in non-feeding animals. Upper Peninsula of Michigan, USA, 2017–2019 (image credit: J. Belant).

We obtained fisher and marten images incidentally at winter carcass sites designed to estimate bobcat (*Lynx rufus*) density^42,43^. We selected 52 sites within hexagonal grid cells of 5.0 km^2^, with the same sites used each year (Fig. 1). Sites were in preferred winter bobcat habitat, typically lowland coniferous forest and riparian areas^59^. As martens and fishers also use lowland conifer and riparian areas^15,17^, we considered our site selection appropriate for this study. We constructed a barrier of woody vegetation at each site to deter wolves and coyotes, leaving four entrances into each site and placed a modified break-away snare at each entrance to collect bobcat hair samples^42^. We baited sites with partial white-tailed deer (*Odocoileus virginianus*; a rib cage and spinal column from local butchers) or beaver (*Castor canadensis*; a half carcass) carcasses wired to a stake or tree. We lured each site with commercial trapping lure (Skunk Junk, D’Aigles’s Lures, MN, USA) placed 2 m above ground (Fig. 5). We installed an infrared remote camera (Model Trophy Cam HD, Bushnell Corporation, Overland Park, Kansas, USA) at each site about 50 cm above ground and 2–4 m from the carcass. Cameras recorded 1 image for each detection with a 5-min delay between detections.

We placed carcasses and lures at sites each winter during 18–21 December and deployed cameras during 2–8 January. Consequently, every site had an unknown history of use by fishers and martens for 2–2.5 weeks before sampling began. After camera deployment, sites were rebaited as needed (generally if the carcass was > 50% depleted) and lure was reapplied every 7 days for 8 occasions during 2 January–5 March. At each visit we downloaded images and replaced batteries as needed. Identification of species in images was performed by the authors and trained technicians, with every marten or fisher image confirmed by at least 2 observers. Fishers and martens were only observed with remote cameras and were not directly involved in the study through capture, tissue samples, or behavioral manipulation; however, our protocols for collecting bobcat hair were approved through Institutional Animal Care and Use Committees through Mississippi State University (IACUC 12-012, 15-013, 17-119) and State University of New York College of Environmental Science and Forestry (IACUC 180501). All methods were carried out in accordance with relevant guidelines and regulations.

### Defining interference competition encounters

To test our prediction that fishers would displace martens at carcass sites but martens would not displace fishers, we defined interference encounter events as cases where one species was displaced by the other at a carcass within 10 min. We chose 10 min to reflect a period where it was likely at least one species was aware of the proximity of the other, while allowing for 1 unidentifiable image of a fleeing or approaching animal (due to the 5-min delay in camera detections). We considered visits independent when at least 30 min had elapsed without detection of the same species at that site^60^. To investigate marten carcass use patterns immediately before and after fisher arrival at carcasses, we tabulated a histogram of marten image counts binned at 5-min intervals within 2 h before and after fisher use of sites.

### Vigilance analysis

For each fisher or marten image, we classified behaviors as: feeding (chewing, sniffing, or head inserted into a cavity within the carcass irrespective of head position), vigilant (eyes above the top of the back at the shoulders but not feeding), and other non-vigilant (not feeding and eye below the back at the shoulders). We excluded images from analysis if the posture was not visible or we were uncertain whether the animal was feeding.

For our response variable in vigilance analyses we coded each observation with a vigilant state as 1 and coded feeding and other non-vigilant states as 0. Because we did not place cameras on carcasses until 2–8 January, we excluded observations during 2–22 January from vigilance regression such that we had a minimum of 14 days of camera monitoring at each site to quantify fisher and marten use. For each species (marten and fisher), we fit a mixed-effects logistic regression model predicting vigilance state (0 or 1) within the package *lme4*^61^ for Program R^62^. We included 3 fixed effects and 1 random effect in each model. The first fixed covariate for both species was days since 1 January within each year, at the time of the image. This covariate was used to estimate temporal changes in vigilance since site establishment due to potential site familiarity and changes in individual condition through winter. The second fixed covariate for both species was daily snow depth within the study area, estimated at the study area centroid by the National Snow and Ice Data Center Snow Data Assimilation System^63^. The brush walls around our sites became taller and more opaque when covered with snow, so we predicted that deep snow may inhibit peripheral vision of feeding animals, necessitating greater vigilance (e.g., standing on hind legs to scan over brush walls). We selected snow depth and days since 1 January as control variables because our covariates of interest used a before and after design within years (see next paragraph), so baseline changes in fisher or marten vigilance correlated with progression of winter could confound interpretation of our results.

The third fixed covariate for marten and fisher vigilance regression was a factor assigned to each image, consisting of four classes indexing previous site use within each year by the other species. Our basis for measuring previous site visitation as a potential risk signal was that mustelids use scent-marking as their primary communication^64,65^, so fishers and martens using carcasses likely had some knowledge of previous use by the other species. Using marten as the example species, we indexed marten or fisher use within the other species vigilance model as follows: (1) no fishers were observed at the site within that year; (2) fishers were observed at the site that year but not within the previous 24 h, (3) low fisher use of that site within 24 h, or (4) high fisher use of that site within 24 h. We classified low and high 24-h fisher site use based on the number of images recorded. To do this, we compiled all marten images with at least one fisher detection within the previous 24 h. We considered image counts greater than the median as high fisher use and image counts equal to or less than the median as low fisher use. We selected 24 h as recent because we considered this a plausible period to expect residual fisher scent to remain yet a long enough period to have enough marten images to fit a precise model. However, as residual mustelid scent can be detected by other species for more than 24 h (e.g., 1 week^66^), we also considered previous mustelid use within the same winter but not within the previous 24 h as potentially influential. Vigilance models for both species included survey year as a random effect to account for possible annual variation in baseline vigilance due to factors we did not quantify.

### Circadian activity analysis

To assess whether martens and fishers partition carcasses temporally and if martens alter diel activity at carcasses in response to fisher presence, we fit 3 circular kernel density estimates of diel activity and compared diel activity kernel overlap and simulated confidence intervals using 1000 iterations within package Activity^67^ for Program R. We fit 2 activity estimates for martens; one for the subset of marten images where a fisher was detected at least once at that site previously during the same winter, and another for the subset of marten images where fishers were not detected previously at that site during the same winter. We then fit an activity estimate using all fisher images for comparison with marten activity. As with vigilance regression, we excluded observations from before 22 January from circadian activity analysis such that we had a minimum of 14 days of monitoring for previous fisher use.

## Data Availability

The datasets used within this study are available from the corresponding author on reasonable request.
